# Electronic Health Record–Based Recruitment and Retention and Mobile Health App Usage: Multisite Cohort Study

**DOI:** 10.2196/34191

**Published:** 2022-06-10

**Authors:** Janelle W Coughlin, Lindsay M Martin, Di Zhao, Attia Goheer, Thomas B Woolf, Katherine Holzhauer, Harold P Lehmann, Michelle R Lent, Kathleen M McTigue, Jeanne M Clark, Wendy L Bennett

**Affiliations:** 1 Department of Psychiatry and Behavioral Sciences Johns Hopkins University School of Medicine Baltimore, MD United States; 2 Welch Center for Prevention, Epidemiology and Clinical Research Johns Hopkins University Baltimore, MD United States; 3 Division of General Internal Medicine Department of Medicine Johns Hopkins University School of Medicine Baltimore, MD United States; 4 Department of Health Policy and Management Bloomberg School of Public Health Johns Hopkins University Baltimore, MD United States; 5 Department of Physiology Johns Hopkins University School of Medicine Baltimore, MD United States; 6 School of Professional and Applied Psychology Philadelphia College of Osteopathic Medicine Philadelphia, PA United States; 7 Division of General Internal Medicine University of Pittsburgh Pittsburgh, PA United States

**Keywords:** mHealth, mobile apps, recruitment, engagement, retention, timing of eating, timing of sleep, obesity, EHR

## Abstract

**Background:**

To address the obesity epidemic, there is a need for novel paradigms, including those that address the timing of eating and sleep in relation to circadian rhythms. Electronic health records (EHRs) are an efficient way to identify potentially eligible participants for health research studies. Mobile health (mHealth) apps offer available and convenient data collection of health behaviors, such as timing of eating and sleep.

**Objective:**

The aim of this descriptive analysis was to report on recruitment, retention, and app use from a 6-month cohort study using a mobile app called Daily24.

**Methods:**

Using an EHR query, adult patients from three health care systems in the PaTH clinical research network were identified as potentially eligible, invited electronically to participate, and instructed to download and use the Daily24 mobile app, which focuses on eating and sleep timing. Online surveys were completed at baseline and 4 months. We described app use and identified predictors of app use, defined as 1 or more days of use, versus nonuse and usage categories (ie, immediate, consistent, and sustained) using multivariate regression analyses.

**Results:**

Of 70,661 patients who were sent research invitations, 1021 (1.44%) completed electronic consent forms and online baseline surveys; 4 withdrew, leaving a total of 1017 participants in the analytic sample. A total of 53.79% (n=547) of the participants were app users and, of those, 75.3% (n=412), 50.1% (n=274), and 25.4% (n=139) were immediate, consistent, and sustained users, respectively. Median app use was 28 (IQR 7-75) days over 6 months. Younger age, White race, higher educational level, higher income, having no children younger than 18 years, and having used 1 to 5 health apps significantly predicted app use (vs nonuse) in adjusted models. Older age and lower BMI predicted early, consistent, and sustained use. About half (532/1017, 52.31%) of the participants completed the 4-month online surveys. A total of 33.5% (183/547), 29.3% (157/536), and 27.1% (143/527) of app users were still using the app for at least 2 days per month during months 4, 5, and 6 of the study, respectively.

**Conclusions:**

EHR recruitment offers an efficient (ie, high reach, low touch, and minimal participant burden) approach to recruiting participants from health care settings into mHealth research. Efforts to recruit and retain less engaged subgroups are needed to collect more generalizable data. Additionally, future app iterations should include more evidence-based features to increase participant use.

## Introduction

Obesity and its related medical comorbidities are highly prevalent public health conditions [1-5]. The strongest evidence for preventing and treating obesity targets health behaviors to modify dietary composition, reduce calories, and increase physical activity [6-8]. Although reducing calories and increasing physical activity result in short-term weight loss, there is a need to identify lifelong behavioral patterns that promote longer-term weight loss and maintenance of healthy weight [9-11]. Aligning the timing of eating and sleeping with intrinsic circadian rhythm (ie, a shorter duration of eating, often called time-restricted eating or feeding) has not yet been thoroughly examined in population-based studies, but has the potential to provide a new paradigm to prevent obesity and metabolic conditions [12-15].

Mobile devices and mobile health (mHealth) apps are ubiquitous, readily available approaches to collect real-time data on health behaviors, such as dietary intake, physical activity, and sleep [16-18]. mHealth apps are often designed and marketed to provide behavioral tracking and lifestyle modification support [19-22]. They also provide a convenient and efficient method for collecting information to advance knowledge about the relationship between obesity-related behavioral patterns and health outcomes [23-25].

Although mHealth research has grown exponentially in the last few decades, study attrition is a major problem, and there is a need to identify successful, low-burden, and efficient recruitment and retention strategies [26,27]. The era of COVID-19, in particular, has additionally highlighted the importance of remote research procedures. Electronic health record (EHR)–based recruitment strategies provide potentially efficient (ie, low touch and low participant burden) methods for identifying and recruiting high volumes of patients meeting predetermined medical criteria for population-based research studies [28-30].

This study presents a secondary analysis from a 6-month, multisite, cohort study that used the EHR to identify and recruit participants to use a mobile app (Daily24), designed to assess timing of eating and sleep [31]. The main goal of the parent observational study was to evaluate the longitudinal association between timing of eating and weight changes over time. Because of the growing interest in both EHR-based recruitment strategies and mHealth data collection methods [19,32-34], the goal of this descriptive analysis is to do the following:

1. Describe the EHR-based recruitment and electronic consent (e-consent) methods and response rates for enrolling in the study and downloading the mobile app.
2. Describe engagement strategies, app use, and retention rates during the 6-month study.
3. Evaluate demographic and behavioral predictors of Daily24 app use.

We hypothesized that people who are younger, have greater education, and have higher BMIs would be more likely to use the app. This study has the potential to inform the field of behavioral health in methodology, uptake, and engagement of mHealth approaches for observational research.

## Methods

### Recruitment

We recruited a cohort of adult patients from three health care systems in the PaTH Clinical Research Network, part of PCORnet (National Patient-Centered Research Network). The three health care systems included the Johns Hopkins Health System, the Geisinger Health System, and the University of Pittsburgh Medical Center [35-37].

### Ethics Approval

Institutional Review Board (IRB) approval was obtained from the Johns Hopkins School of Medicine (IRB00174516), which had a reliance agreement with the other institutions’ IRBs.

### EHR-Based Participant Eligibility Criteria

Potential participants were identified using EHR-based eligibility criteria (ie, “computable phenotype” [38]) to query the EHR. Each site also obtained a list of potentially eligible participants who previously consented to complete PaTH cohort studies at these sites [37]. Eligibility criteria included the following: at least 18 years of age and a minimum of one weight measurement and one height measurement recorded in the EHR between July 2017 and July 2019. Participants were excluded if they were deceased.

### Recruitment Messaging Via Email and the Patient Portal

Potentially eligible participants were sent recruitment messages via email or the patient portal (ie, Epic MyChart) from February to July 2019. Each partnering health care system tailored its own strategy to recruit participants from the large pool of potentially eligible patients who were identified using the computable phenotype. One site used patient portal recruitment almost exclusively, focusing on patients who had a health system visit in the last week. The other two sites sent email recruitment letters through their primary care and weight management practices, with messages signed by the clinic medical directors. Multimedia Appendix 1 shows a sample recruitment message, which included a brief study description and link to a web-based e-consent form.

### e-Consent and Enrollment Process

**Figure 1:**
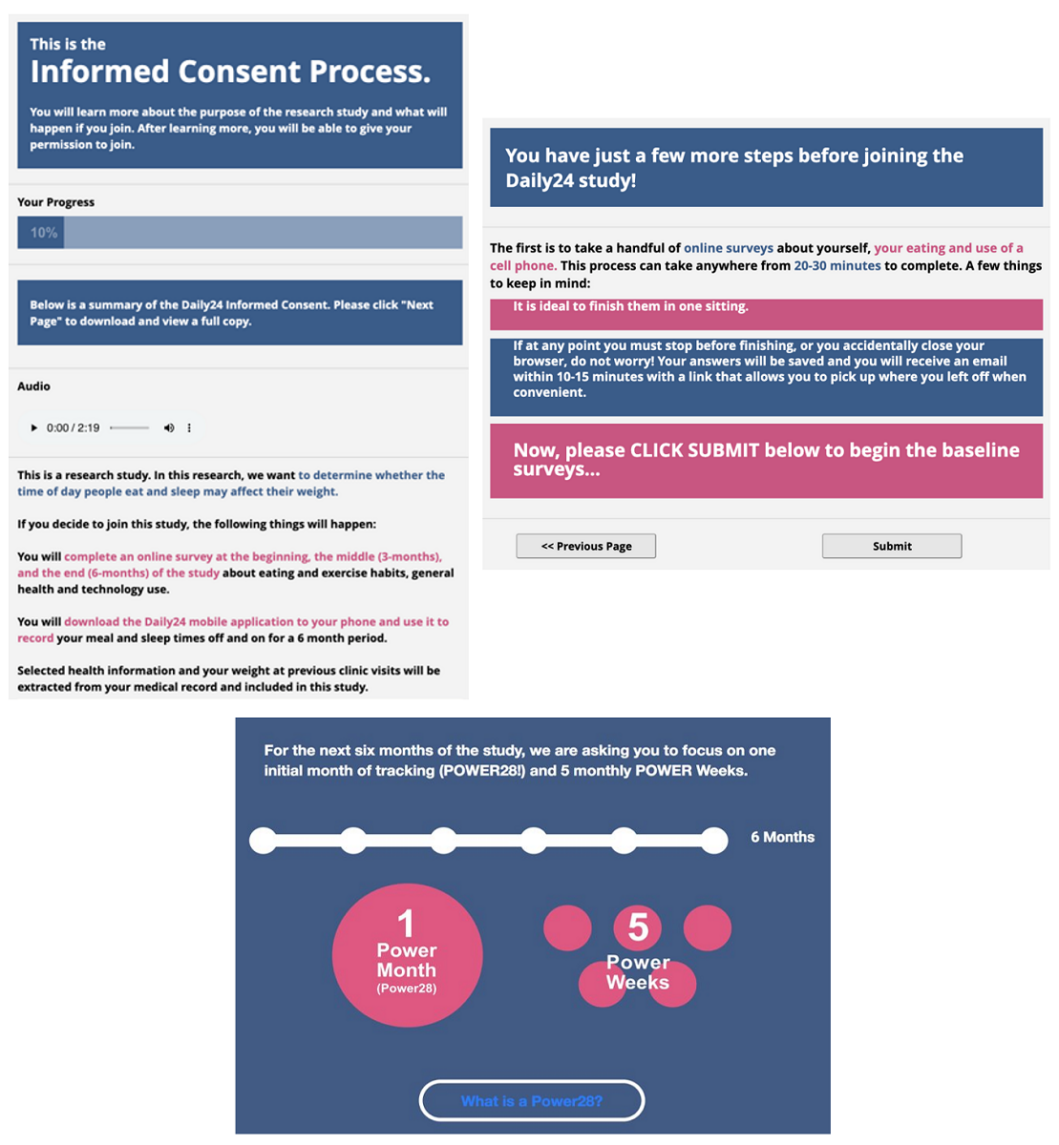
Screenshots of web-based electronic recruitment and onboarding: electronic consent (top left), baseline surveys (top right), and POWER 28 and POWER week information (bottom).

We designed a web-based e-consent process in REDCap (Research Electronic Data Capture) beginning with a study description, including participant expectations and duration (see Figure 1). Upon confirming interest, participants proceeded to the e-consent form, which included a supplemental audio clip of the consent form being read aloud, followed by a short quiz to ensure comprehension. Consenting participants provided identifying information (ie, full name, date of birth, and email), enabling staff to link each participant to the EHR for future analyses (not reported herein). Once consented, participants received a link to complete online baseline surveys (Figure 1). Participants were considered enrolled in the cohort after completing baseline surveys, at which time they received instructions on how to download and use the Daily24 mobile app.

### Daily24 Mobile App, Registration, and Download

**Figure 2:**
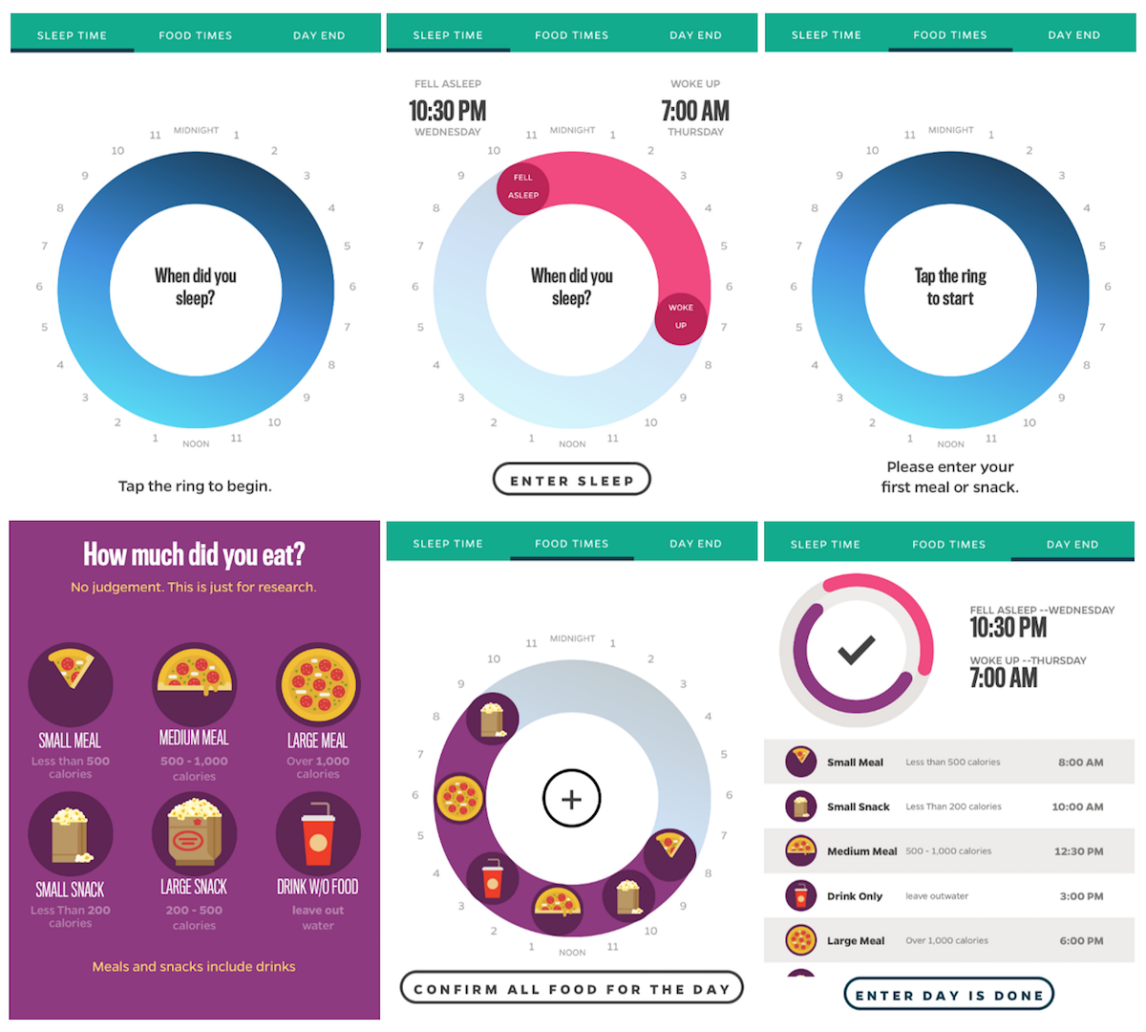
Screenshots of the Daily24 app: empty sleep ring (top left), complete sleep ring (top middle), empty food ring (top right), meal size selection (bottom left), complete food ring (bottom middle), and complete day (bottom right).

The Daily24 mobile app was custom designed by our research team to collect information from participants about the timing of eating and sleep, including wake time, sleep time, timing of each eating occasion, and estimate of amount eaten (ie, small, medium, or large meal; small or large snack; or drink, except water, without food) during a 24-hour window (Figure 2). The design of the app is described elsewhere [31]. We benefited from the input of patient and end-user stakeholders in the design of the mobile app, as well as recruitment and retention methods, and we pilot-tested the app [31,39].

Following enrollment in the cohort, participants received a text message on their mobile phones with a unique link to the Daily24 registration form. This unique link contained a token (ie, 11-character universally unique identifier) that enabled the study team to connect participants’ data between the mobile app and their online enrollment information and surveys, while preserving privacy. Registration included an overview of how to use the app, study timeline, and incentives (see next section), followed by selection of a unique “Daily24 name” from a list of randomly generated combined nouns (eg, “FloatHarbor”) that, once selected, was the participant’s Daily24 username. Participants then received a link to download the Daily24 app via iOS (Apple Store) or Android (Google Play).

### Engagement Strategies to Promote Use of the Daily24 Mobile App

**Figure 3:**
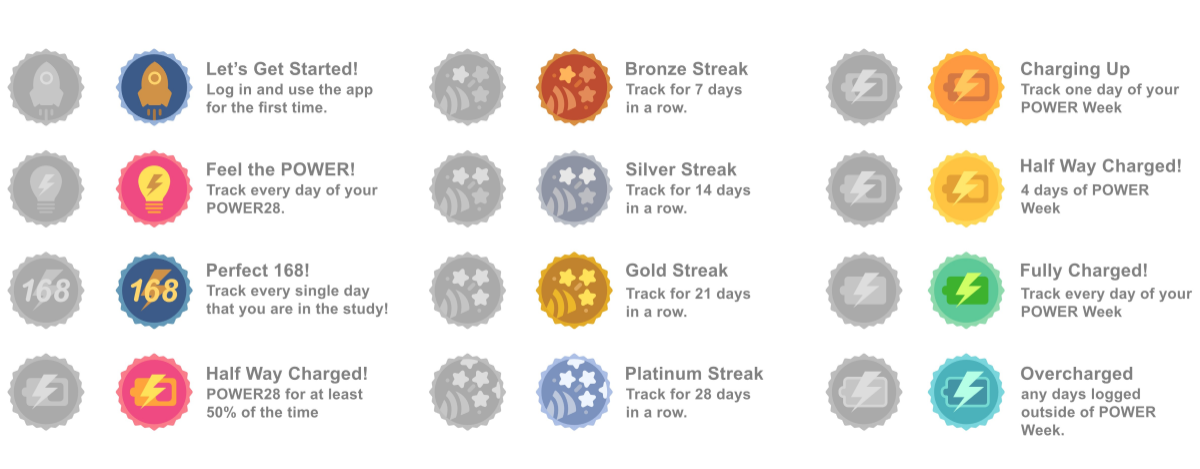
Screenshots of the badges earned to encourage usage in the Daily24 app.

Although we encouraged participants to enter as much data as possible over the 6-month study, we developed and applied strategies aimed at maximizing app use during their first 4 weeks of participation (ie, 28 days after downloading the app, called “POWER 28”) and 1 week per month for the remaining 5 months of the study, called “POWER weeks” (Figure 1). These highly targeted usage days for the study were equivalent to 63 days (POWER 28 + POWER weeks × 5 weeks). Engagement strategies included a leaderboard, badges, raffles, and text reminders. The leaderboard displayed the number of consecutive days tracked on one tab (ie, streak) and total number of all days tracked on the other tab. Earned badges encouraged various types of app use, including one-time badges, streak badges, and POWER week badges (Figure 3). We raffled off US $25 gift cards weekly throughout the study, with those earning more badges having greater odds of winning the raffle. We used emails, SMS text messages, and in-app notifications to encourage usage and to remind participants where they were in their POWER 28 and when a POWER week was coming up. The logic for these messages was triggered both by time (ie, close to a POWER week) as well as by lack of a response (ie, an event missing data). If a participant was on track with logging events, we simply encouraged their continued involvement and did not send additional reminders.

### Data Collection

Daily24 app usage data were collected using Amazon Web Services. Self-reported online surveys were administered using REDCap at baseline and at the end of 4 months using standard measures assessing demographics, mHealth use, height, and weight, as well as eating, physical activity, and sleep habits. Technology use and health app use were assessed using the Pew Social Media Update 2016 [40] and a survey measuring characteristics of health app use [16], respectively. Nutritional and eating assessments included the National Health and Nutrition Examination Survey 2009-2010 Dietary Screener Questionnaire [41], which provided estimates of fruit, vegetable, and sugar-sweetened beverage intake over the last 30 days. Physical activity was assessed using the self-administered, short version of the International Physical Activity Questionnaire [42]; physical activity levels were categorized as low, medium, or high over the last 7 days. Sleep measurements included the single sleep quality item from the Pittsburgh Sleep Quality Index [43] and study-created questions about frequency of daytime naps.

To facilitate and encourage baseline survey completion, participants received automated reminders at 15 minutes, 24 hours, and 48 hours after consent and had up until 90 days after receiving the initial survey link to complete the survey. Personalized survey-engagement strategies included a combination of staff emails, text messages, and US $100 raffle gift cards. Participants had up until 60 days to complete the 4-month measures, but this paper only reports baseline survey descriptive results. Data collection was completed in January 2020.

### App Usage Categories

App users were defined as using the Daily24 app for at least one day, which was captured by having at least one meal and sleep entry, on at least one day, and pushing “done for the day” on the screen. Nonusers either did not register or download the app or did not push “done for the day” on any day. App use was further categorized into three non–mutually exclusive ways:

1. Immediate use, defined as using the app for 7 days or more during the POWER 28.
2. Consistent use, defined as using the app for 28 days or more during the entire 6-month study, which was based on using the app equal to or more than the median overall days of use for the entire 6-month study.
3. Sustained use, defined as using the app for at least 2 days during the last POWER week (month 6) of the study.

### Statistical Analyses

This was a secondary analysis of data from a parent cohort study. We used descriptive statistics (Student t tests or χ^2^ tests) for baseline characteristics for all participants and by app use versus nonuse categories. App use was also described in median days of use for the total study, median days used in targeted and nontargeted usage days, and frequency of 2 or more and 7 or more days of use during each month of the study. We selected these two categories based on the following logic:

1. Two or more days: this was selected to represent a low threshold of app use that was not identical to the minimal definition of being an app user.
2. Seven or more days: this was selected because we focused on POWER weeks during months 2 to 6 and wanted to capture those who achieved at least one week of usage.

We evaluated the association between baseline characteristics, with app usage as the dependent variable, using multivariable logistic regression models. Multivariable logistic regression was also used to model the association between baseline characteristics with immediate, consistent, and sustained app use. We used two models with progressive adjustment. Model 1 adjusted for key demographics, including age, sex, race, education, household income, and children younger than 18 years old. Model 2 additionally adjusted for key behavioral factors that could influence engagement, including physical activity, fruit and vegetable servings, sleep quality, and BMI. Covariates were nonmissing, prespecified, and based on a priori hypotheses.

## Results

### Enrollment and App Use

**Figure 4:**
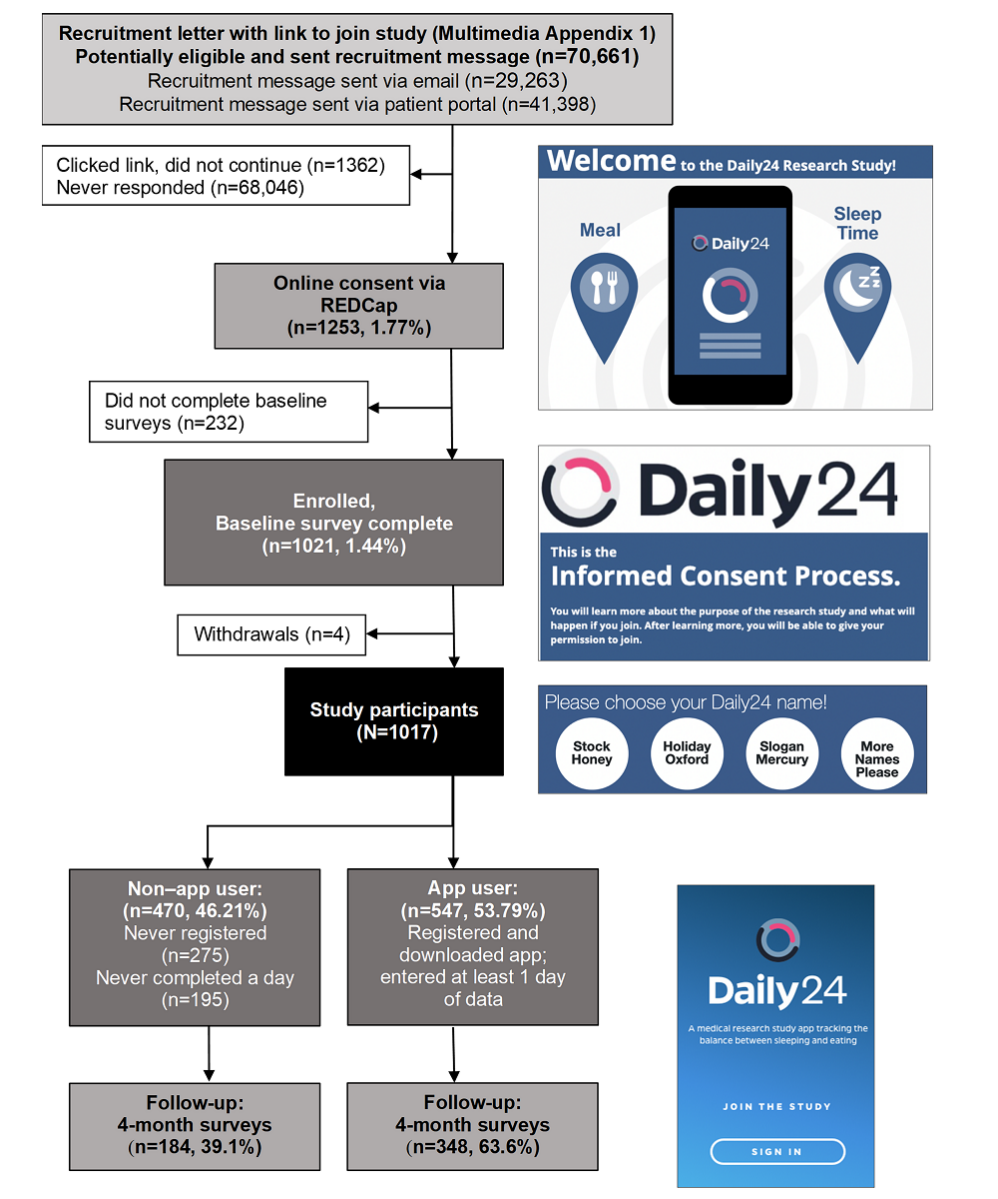
Recruitment and retention flow. REDCap: Research Electronic Data Capture.

Figure 4 shows the enrollment flow of eligible participants. Electronic recruitment messages were sent to 70,661 potentially eligible participants, with 1253 participants (1.77%) completing the e-consent process and 1021 (1.44%) enrolling by completing baseline surveys. A total of 4 participants withdrew, leaving 1017 participants included in the analytic sample. Participant characteristics are reported in Table 1. The majority of the 1017 participants were female (n=790, 77.68%), White (n=788, 77.48%), and college graduates (n=749, 73.65%), and the mean age was 51.1 (SD 15.0) years.

**Table 1:** Description of study participants and potential confounders at baseline.

Characteristics and confounders		All participants (N=1017)	Non–app users (n=470)	App users^a^ (n=547)	*P* value^b^
Age (years), mean (SD)		51.1 (15.0)	53.2 (14.6)	49.3 (15.0)	<.001
**Gender, n (%)**					
	Male	224 (22.0)	115 (24.5)	109 (19.9)	.07
	Female	790 (77.7)	355 (75.5)	435 (79.5)	
	Prefer not to answer	3 (0.3)	0 (0)	3 (0.5)	
**Race, n (%)**					
	White	788 (77.5)	351 (74.7)	437 (79.9)	.14
	Black	149 (14.7)	82 (17.4)	67 (12.2)	
	Asian	29 (2.9)	13 (2.8)	16 (2.9)	
	Pacific Islander, American Indian, or others	17 (1.7)	10 (2.1)	7 (1.3)	
	Two or more races	34 (3.3)	14 (3.0)	20 (3.7)	
**Site, n (%)**					
	Site A	51 (5.0)	23 (4.9)	28 (5.1)	.004
	Site B	282 (27.7)	105 (22.3)	177 (32.4)	
	Site C	200 (19.7)	96 (20.4)	104 (19.0)	
**Educational level, n (%)**					
	High school or less	63 (6.2)	40 (8.5)	23 (4.2)	<.001
	Some college	205 (20.2)	109 (23.2)	96 (17.6)	
	College graduate	749 (73.6)	321 (68.3)	428 (78.2)	
**Annual household income (US $), n (%)**					
	<35,000	120 (11.8)	70 (14.9)	50 (9.1)	.02
	35,000 to <50,000	109 (10.7)	53 (11.3)	56 (10.2)	
	50,000 to <75,000	148 (14.6)	66 (14.0)	82 (15.0)	
	≥75,000	550 (54.1)	234 (49.8)	316 (57.8)	
	Don’t know/choose not to answer	90 (8.8)	47 (10.0)	43 (7.9)	
Any child <18 years old, n (%)		248 (24.4)	129 (27.4)	119 (21.8)	.04
Height (cm), mean (SD)		168.9 (50.2)	170.6 (73.3)	167.4 (8.5)	.31
Weight (kg), mean (SD)		85.8 (23.8)	86.8 (25.1)	85.0 (22.5)	.23
BMI^c^, mean (SD)		30.5 (7.9)	30.8 (8.2)	30.3 (7.6)	.29
**BMI categories, n (%)**					
	Underweight (<18.5)	14 (1.4)	7 (1.5)	7 (1.3)	.75
	Normal (18.5 to <25)	250 (24.6)	111 (23.6)	139 (25.4)	
	Overweight (25 to <30)	288 (28.3)	129 (27.4)	159 (29.1)	
	Obese (≥30)	465 (45.7)	223 (47.4)	242 (44.2)	
Fruit or vegetable cup equivalent, mean (SD)		2.9 (1.5)	2.8 (1.6)	3.0 (1.4)	.18
Added sugars tsp equivalent from sugar-sweetened beverages, mean (SD)		0.8 (1.3)	1.0 (1.5)	0.7 (1.2)	.004
**Physical activity, n (%)**					
	Low	20 (5.1)	11 (6.3)	9 (4.1)	.53
	Medium	221 (55.8)	94 (53.7)	127 (57.5)	
	High	155 (39.1)	70 (40.0)	85 (38.5)	
**Sleep quality, n (%)**					
	Very good	188 (18.5)	76 (16.2)	112 (20.5)	.02
	Fairly good	496 (48.8)	222 (47.2)	274 (50.1)	
	Fairly bad	274 (26.9)	136 (28.9)	138 (25.2)	
	Very bad	59 (5.8)	36 (7.7)	23 (4.2)	
**Nap frequency, n (%)**					
	<1 per week	581 (57.1)	267 (56.8)	314 (57.4)	.03
	1 per week	165 (16.2)	69 (14.7)	96 (17.6)	
	2-3 per week	176 (17.3)	79 (16.8)	97 (17.7)	
	4-6 per week	57 (5.6)	28 (6.0)	29 (5.3)	
	Daily	38 (3.7)	27 (5.7)	11 (2.0)	
**Number of health apps used in past month, n (%)**					
	0	212 (20.8)	127 (27.0)	85 (15.5)	<.001
	1-5	705 (69.3)	299 (63.6)	406 (74.2)	
	>5	100 (9.8)	44 (9.4)	56 (10.2)	
**App use reasons, n (%)**					
	Track how much exercise I get	665 (65.4)	269 (57.2)	396 (72.4)	<.001
	Track what I eat/improve what I eat	531 (52.2)	212 (45.1)	319 (58.3)	<.001
	Weight loss	476 (46.8)	206 (43.8)	270 (49.4)	.08
	Track a health measure	203 (20.0)	86 (18.3)	117 (21.4)	.22
	Track how much sleep I get	346 (34.0)	132 (28.1)	214 (39.1)	<.001

^a^App user is defined as downloading the app and recording at least one entry on at least one day.

^b^The *P* value for a group of variables is reported in the row of the first variable.

^c^BMI is calculated as weight in kilograms divided by height in meters squared.

Out of 1017 participants, 547 (53.79%) were app users (ie, downloaded the app and recorded at least one entry on at least one day). When examining app users by use category, 412 (75.3%), 274 (50.1%), and 139 (25.4%) were categorized as immediate, consistent, and sustained users, respectively. Of the sustained users, 116 (83.5%) used the app at least one day every month of the study, and 133 (95.7%) used the app at least one day for 5 out of the 6 months. In comparison to non–app users (471/1017, 46.31%), app users were younger (mean 49.3 vs 53.3 years; *P*<.001), more likely to be college graduates (78.2% vs 68.3%; *P*<.001), had greater annual income (>US $50,000: 398/547, 72.8% vs 300/470, 63.8%; *P*=.02), and were less likely to have children younger than 18 years old (21.8% vs 27.4%; *P*=.04). There were no differences between app users and nonusers regarding weight, height, mean BMI, and BMI category. App users were less likely to drink sugar-sweetened beverages (mean sugar tsp equivalent: 0.7 vs 1.0; *P*=.004), reported better sleep quality (fairly good or very good: 386/547, 70.6% vs 298/470, 63.4%; *P*=.02), and were less likely to take daily naps (2.0% vs 5.7%; *P*=.03). They were also more likely to use health apps overall (462/547, 84.5% vs 343/470, 73.0%; *P*<.001), and to use them for the purpose of tracking exercise (396/547, 72.4% vs 269/470, 57.2%), eating (319/547, 58.3% vs 212/470, 45.1%), and sleep (214/547, 39.1% vs 132/470, 28.1%; *P*<.001 for all).

The median amount of app use was 28 (IQR 7-75) days over the 6-month study, 20 (IQR 7-35) days during the targeted 63 days of the study, and 6 (IQR 0-41) days during the 117 nontargeted days of the study. Table 2 describes app use by study month. During study month 1, the vast majority of app users (92.3%) used the app for 2 or more days and 76.2% used it for 7 or more days. Usage decreased over time in the cohort study. By month 6, 27.1% of app users used the app for 2 or more days and 20.1% used it 7 or more days.

**Table 2:** Monthly Daily24 app use during the 6-month cohort study by users who completed at least one day of app use.

Month^a^	Participants who used the app (n=547), n (%)	
	Used ≥2 days	Used ≥7 days
Month 1	505 (92.3)	417 (76.2)
Month 2	269 (49.2)	214 (39.1)
Month 3	213 (38.9)	166 (30.3)
Month 4	183 (33.5)	138 (25.2)
Month 5^b^ (n=536)	157 (29.3)	133 (24.8)
Month 6^b^ (n=527)	143 (27.1)	106 (20.1)

^a^A study month is defined as 4 weeks (28 days). To enable all study months to begin on a Monday, the time between the end of POWER 28 and start of month 2 ranged from 15 to 21 days.

^b^Due to late registration, some participants were not able to reach months 5 and 6 of the study.

### Predictors of Usage of the Daily24 App

Table 3 shows the multivariable regression model for app use versus nonuse. Younger age, White (vs non-White) race, greater education, higher household income, not having children less than 18 years of age, and having used 1 to 5 apps in the past were statistically significantly associated with app use (vs non–app use). Black participants were one-third less likely to use the app than White participants, whereas those with greater than a college education and a higher income (≥US $75,000 vs <US $35,000) were statistically significantly more likely to use the app. Those with children under the age of 18 years were 45% less likely to use the app, and those who had used 1 to 5 apps in the past month were 70% more likely to use the app compared to those who had not used apps in the past month.

Table 4 shows multivariable regression models for immediate, consistent, and sustained use. Older age and lower BMI were statistically significantly associated with increased immediate, consistent, and sustained app use. Having children less than 18 years old was statistically significantly associated with decreased immediate use, and better sleep quality was associated with increased immediate and consistent app use.

**Table 3:** Multivariable regression models for Daily24 app use versus nonuse.

Risk factors				Model 1^a^				Model 2^b^		
				OR^c^ (95% CI)		*P* value		OR (95% CI)		*P* value
**Demographic risk factors**										
	Age, per 10-year increase			0.77 (0.70-0.85)		<.001		0.78 (0.71-0.86)		<.001
	**Gender**									
		Male	Ref^d^ (1)				Ref (1)			
		Female	1.32 (0.96-1.81)		.09		1.22 (0.88-1.69)		.23	
	**Race**									
		White	Ref (1)				Ref (1)			
		Black	0.66 (0.45-0.96)		.03		0.67 (0.46-0.98)		.04	
		Other	0.80 (0.49-1.31)		.38		0.82 (0.50-1.34)		.43	
	**Educational level**									
		<College	Ref (1)				Ref (1)			
		≥College	1.39 (1.03-1.89)		.03		1.36 (1.00-1.86)		.05	
	**Household income (US $)**									
		<35,000	Ref (1)				Ref (1)			
		35,000 to <50,000	1.58 (0.91-2.72)		.10		1.40 (0.80-2.44)		.24	
		50,000 to <75,000	2.01 (1.20-3.38)		.01		1.82 (1.07-3.07)		.03	
		≥75,000	2.30 (1.47-3.61)		<.001		2.00 (1.26-3.17)		.003	
	**Any child <18 years old**									
		No	Ref (1)				Ref (1)			
		Yes	0.53 (0.39-0.73)		<.001		0.55 (0.40-0.75)		<.001	
**Behavioral risk factors**										
	**Physical activity**									
		Low or medium	—^e^		—		Ref (1)			
		High	—		—		0.93 (0.61-1.43)		.75	
	Fruit and vegetable cups, per 1-cup increase			—		—		1.04 (0.95-1.14)		.41
	**Sleep quality**									
		Very good or fairly good	—		—		Ref (1)			
		Very bad or fairly bad	—		—		0.79 (0.59-1.04)		.09	
	**Number of health apps used in past month**									
		0	—		—		Ref (1)			
		1-5	—		—		1.70 (1.22-2.37)		.002	
		>5	—		—		1.40 (0.84-2.35)		.20	
	BMI^f^, per 1-unit increase			—		—		1.00 (0.98-1.02)		.99

^a^Model 1 was adjusted for age, sex, race, education, household income, and having children younger than 18 years old.

^b^Model 2 included model 1 parameters and was adjusted for physical activity, fruit and vegetable cups, sleep quality, and BMI.

^c^OR: odds ratio.

^d^Ref: reference.

^e^Not calculated since these parameters were not included in model 1.

^f^BMI is calculated as weight in kilograms divided by height in meters squared.

**Table 4:** Multivariable regression models^a^ for immediate, consistent, and sustained Daily24 app use (n=547).

Risk factors				Immediate use: using app for ≥7 days during POWER 28 (n=412)					Consistent use: using app for ≥28 days for 6 months (n=274)					Sustained use: using app for ≥2 days during POWER week 5 (n=139)		
				OR^b^ (95% CI)		*P* value		OR (95% CI)			*P* value		OR (95% CI)			*P* value
**Demographic risk factors**																
	Age, per 10-year increase			1.28 (1.09-1.50)		.003		1.40 (1.22-1.61)			<.001		1.54 (1.31-1.82)			<.001
	**Gender**															
		Male	Ref^c^ (1)				Ref (1)					Ref (1)				
		Female	0.69 (0.38-1.26)		.23		0.60 (0.37-0.98)			.04		0.74 (0.44-1.25)			.26	
	**Race**															
		White	Ref (1)				Ref (1)					Ref (1)				
		Black	0.70 (0.38-1.30)		.26		0.86 (0.48-1.52)			.60		0.92 (0.46-1.84)			.82	
		Other	0.79 (0.38-1.66)		.53		0.67 (0.33-1.36)			.27		1.03 (0.45-2.38)			.94	
	**Educational level**															
		<College	Ref (1)				Ref (1)					Ref (1)				
		≥College	1.00 (0.59-1.71)		.99		0.99 (0.61-1.59)			.96		1.46 (0.82-2.60)			.20	
	**Household income (US $)**															
		<35,000	Ref (1)				Ref (1)					Ref (1)				
		35,000 to <50,000	0.94 (0.38-2.29)		.89		1.05 (0.46-2.42)			.91		0.86 (0.31-2.37)			.77	
		50,000 to <75,000	0.83 (0.36-1.92)		.66		1.18 (0.54-2.56)			.68		0.87 (0.35-2.16)			.76	
		≥75,000	1.00 (0.47-2.14)		.99		0.76 (0.38-1.53)			.44		0.62 (0.27-1.41)			.25	
	**Any child <18 years old**															
		No	Ref (1)				Ref (1)					Ref (1)				
		Yes	0.56 (0.34-0.91)		.02		0.68 (0.43-1.07)			.10		0.70 (0.38-1.28)			.24	
**Behavioral risk factors**																
	**Physical activity**															
		Low or medium	Ref (1)				Ref (1)					Ref (1)				
		High	0.68 (0.35-1.32)		.25		1.01 (0.55-1.83)			.98		1.30 (0.68-2.51)			.43	
	Fruit and vegetable cups, per 1-cup increase			0.88 (0.75-1.03)		.10		1.03 (0.90-1.19)			.64		0.98 (0.84-1.15)			.82
	**Sleep quality**															
		Very good or fairly good	Ref (1)				Ref (1)					Ref (1)				
		Very bad of fairly bad	0.59 (0.38-0.93)		.02		0.63 (0.42-0.95)			.03		0.74 (0.45-1.21)			.23	
	**Number of health apps used in past month**															
		0	Ref (1)				Ref (1)					Ref (1)				
		1-5	0.86 (0.45-1.63)		.64		1.12 (0.66-1.92)			.67		1.46 (0.81-2.62)			.20	
		>5	1.03 (0.42-2.51)		.95		1.04 (0.49-2.24)			.91		0.60 (0.21-1.70)			.34	
	BMI^d^, per 1-unit increase			0.96 (0.94-0.99)		.01		0.95 (0.93-0.98)			.001		0.95 (0.92-0.99)			.01

^a^The model was adjusted for age, sex, race, education, household income, having children younger than 18 years old, physical activity, fruit and vegetable cups, sleep quality, and BMI.

^b^OR: odds ratio.

^c^Ref: reference.

^d^BMI is calculated as weight in kilograms divided by height in meters squared.

### Survey Completion and Retention in the EHR-Based Cohort Study

Out of 1017 enrolled participants, 328 (32.25%) completed the 4-month follow-up surveys within 72 hours of receiving the link. Of the remaining 689 participants (67.75%), study staff were able to reach out to 610 participants (88.5%) through personalized emails, text messages, and US $100 raffle invitations delivered up to one week prior to study completion. Of the 610 contacted participants, 113 (18.5%) completed their surveys after one contact, 56 (9.2%) after two contacts, and 35 (5.7%) after three contacts, increasing the overall number of 4-month survey completers to 532 (52.31%).

## Discussion

### Principal Findings

EHRs and patient portals are readily available through most health care systems, and use of mHealth apps is fairly ubiquitous [16,44]. This study reports on EHR-based recruitment of adults from three health systems to use the Daily24 mobile app to record daily timing of meals, snacks, and sleep for 6 months. We emailed research invitations to over 70,000 potentially eligible participants identified through the EHR using efficient identification (ie, computable phenotype) and messaging methods (ie, emails sent directly through the EHR patient portal or to personal email addresses). A total of 1.4% of participants completed e-consent forms and baseline questionnaires in a period of 6 months, a yield that is slightly lower than reports for other EHR-based recruitment methods [32-34]. In a 2019 single-institution study that included 13 separate EHR-based recruitment strategies using the patient portal recruitment service, the average response rate for patient portal messages was 2.9% [32]. Our lower yield might be explained by the study’s expectation to download and actively use an app for 6 months or have no guaranteed compensation be provided for participation [45] (ie, raffles of gift cards). Patients may also be more likely to respond to mHealth research with a behavioral intervention [46] or to disease-related versus wellness-related research [32,45]. In the above study by Miller and colleagues [32], recruitment response rates were higher (3.4%) among condition-specific studies (ie, those with a more inclusive comprehensive phenotype) versus general health studies (1.4%). The latter response rate was identical to this study’s recruitment yield, which was also not specific to a health condition. Furthermore, while our app included gaming elements (eg, badges and a leaderboard) [47,48] to increase data entry, we intentionally did not include behavioral techniques (eg, goal setting and personalized behavioral prompts) aimed at behavior change, given the study’s primary goal to naturalistically examine the relationship between timing of eating and sleep and weight and medical conditions (findings forthcoming).

Once enrolled, 54% of participants who downloaded the app entered timing of eating or sleep data on at least one day. While the frequency criteria to classify someone as an app user in this study was fairly low (ie, at least one completed day), other studies have used a similarly low frequency to define usage [49]; however, comparisons between studies can be challenging due to disparate study designs and modes of interacting with apps (ie, passive vs active data collection) [50]. For example, in the Asthma Mobile Health Study (AMHS), 85.21% (6470/7593) of enrolled participants (ie, downloaded an asthma health app, e-consented, and verified email) were considered baseline users (ie, at least one in-app survey entry). However, enrollment occurred after the app was already downloaded, and individuals who downloaded the app (N=40,683 in the United States over 6 months) were recruited through a large media blitz versus academic recruitment [49]. Eligibility was also based on having a medical condition (ie, disease related), and the app included behavioral components (eg, goal setting).

Although criteria for defining usage categories differ across studies, our immediate (75%; ≥7 days in the first month) and consistent (50%; ≥28 days over 6 months) rates were higher than the “robust” cohort rates (30%; 5 or more surveys over 6 months) reported in the AMHS; in the case of sustained users (25%; ≥2 days during month 6), our rates were fairly comparable to those in the AMHS [49]. We attribute being able to initially engage three-quarters of our app users, and to retain a quarter of our users, to the food and sleep wheels in Daily24 being fast and easy to use, whereas other apps may include more survey items or require more detailed dietary intake entry [31,51]. Future iterations of the app should employ evidence-based strategies and features for increasing engagement (eg, push notifications with tailored health messages) [52-54].

This study’s usage data provides important information about predictors of health app use to guide the design of future observational studies using apps. Our finding that those who were younger, more formally educated, and wealthier were more likely to be app users is consistent with past research [16,49]. This study also found that White participants were more likely to be app users, a finding that is consistent with some research [55]. However, that finding is not consistent with a cross-sectional survey study of 1604 mobile phone users in the United States [16], which found that being Latino or Hispanic (*P*<.05) or African American (*P*=.07, trend) were related to a greater likelihood to download a health app. Inconsistencies in findings may be related to different assessment methods (ie, actual app usage vs self-reported use), recruitment methods (ie, national survey vs regional EHR recruitment), and racial and ethnic distribution in recruitment regions [16,56]. Not having children younger than 18 years of age was also associated with app use. While this is perhaps a correlate of being younger, it is also an intuitive finding that those with children may have less time for mHealth app use, supporting the well-documented importance of ease and efficiency of data entry in mHealth apps [16]. While younger age was associated with app use overall, being older was associated with early, consistent, and sustained use. The AMHS study similarly found that among robust users, increasing age was significantly associated with a greater likelihood to use the asthma health app daily [49]. An adherence and retention study of a web-based alcohol intervention also found that being older and not having children predicted a greater likelihood of logging in [57]. We also found that having a lower BMI was associated with early, consistent, and sustained use. Past research has found that having a BMI in the obese range is associated with greater health app use [16], influencing our hypothesis that those with higher BMIs would be more motivated to download and use a health app. While we did not find a significant association between weight status and app use overall, we did find that those with lower BMIs were more likely to use health apps across time. Given this observational study design, we identified an association between BMI and health app use (ie, those with a lower BMI were more likely to engage in sustained tracking and health monitoring), but we do not know causality or temporality. Future research exploring a causal relationship is needed to determine if apps targeting timing of eating and sleep may have an effect on behaviors that influence weight [25,56].

### Limitations

There are several limitations of this study. First, this was an observational cohort study and was not designed with a comparison group to assess differences in app use among participants instructed to log for 6 months without additional guidance on targeted tracking days, compared to our approach of emphasizing tracking on POWER 28 and POWER week days. In designing the study, to optimize longitudinal tracking, we decided to preidentify targeted days to decrease participant burden and, more importantly, to increase the likelihood that we would collect data on some days across each of the 6 months rather than risk the typical pattern of heavier use up front followed by drop-off [18,49]. This approach appeared to be effective. Although we did observe drop-off across each study month, with the biggest decline being from months 1 and 2, about one-quarter of the participants were still using the app during month 6, and those who were using the app during month 6 were using it in the identified POWER week. However, without a two-arm study design, we cannot fully conclude that this was the ideal approach. Second, although we designed badges and a leaderboard to create gaming elements and increase motivation [47,48], we are unable to ascertain if those who earned badges were more motivated individuals in general or were motivated by the badges. Badges were earned based on various categories of usage (eg, first log-in, track 7 days in a row, and 4 days of your POWER week) and were automatically entered into our raffle (ie, participants did not have to enter their badges into the raffle themselves); thus, it is challenging to know whether badges and the resulting raffle were an effective gamification approach. Third, we do not have detailed information on the reasons that a little less than half of the participants did not go on to download the app. With our app being designed by researchers rather than more highly funded industry, we suspect that the onboarding process may have had some cumbersome features. The biggest obstacles may have been problems with the two-factor authentication process, which required participants to receive an SMS code on their device and correctly enter it to verify their identity. Additionally, many people forgot, misplaced, or mis-entered the password they chose when registering for the app and were without an automated password reset option. Although we had research staff available for tech support, it was available only during work hours and via phone or email. Fourth, our sample was largely comprised of White participants, more formally educated participants, and those of middle- to upper-socioeconomic status; thus, the generalizability to other racial, ethnic, and socioeconomic groups is limited. Future research involving EHR-based recruitment independent of technology use might consider partnering with communities from racialized and lower-socioeconomic subgroups to understand how recruitment efforts and health apps can be adapted to improve their impact for marginalized communities. Finally, while our recruitment methods were efficient in terms of participant identification, messaging, and enrollment, we are unable to comment on the cost-effectiveness of EHR enrollment. Each of these health systems have made significant investments into building and maintaining their EHRs and infrastructure to enable these recruitment methods for research purposes. In addition, for this study, we leveraged existing health information technology infrastructure from the PaTH network [30], which enabled efficiency from both a time and resource perspective. However, for this methodology to be used more broadly in a variety of settings, greater institutional and community partnerships and resources are needed.

### Conclusions

Health apps aimed at weight loss and related behaviors are among the most highly used mHealth apps [22]. Time-restricted feeding is a novel and promising approach for obesity and related disease management; however, it is largely untested in humans, to a great extent due to the challenges of helping individuals modify their behavior to a shorter window of eating [15,58,59]. This report is a first step in describing efficient EHR recruitment of patients from three large health institutions and the use of an mHealth app to enter information about timing of eating and sleep patterns. Next steps include incorporating behavioral techniques into the app, potentially with health coaching, to assist individuals achieve greater alignment with their circadian rhythms and to determine whether this is a feasible and effective weight loss intervention.

## Appendix

Multimedia Appendix 1 Sample recruitment message.

## Abbreviations

AMHS: Asthma Mobile Health Study
e-consent: electronic consent
EHR: electronic health record
IRB: Institutional Review Board
mHealth: mobile health
PCORnet: National Patient-Centered Research Network
REDCap: Research Electronic Data Capture
