# Supplementary material for: Electronic Health Record–Based Recruitment and Retention and Mobile Health App Usage: Multisite Cohort Study
Source: J Med Internet Res. 2022 Jun 10;24(6):e34191. doi: 10.2196/34191 (PMC9233254; doi:10.2196/34191)
Supplement: Multimedia Appendix 1 [file jmir_v24i6e34191_app1.pdf]

SUBJECT: You're Invited to Join the Daily24 Team!

Dear [Patient Name],

Do you wonder how the timing of your meals and sleep impacts your health? So do we!

We are inviting you to join a new research study with our team from [Names of Institutions]. The goal of this study is to understand how timing of eating and sleep affect weight over time.

Our research team at [Name of Institution] has developed a mobile application (app) for your phone called Daily24. The app allows the study team to easily collect information from you about when you eat and sleep. This app is fun, quick and easy to use!

Participation in this study requires keeping track of your meal and sleep times with the Daily24 app off and on for 6 months. You will also allow the team to review your medical records. We are sending this opportunity to all patients at [Name of Institution] who might be interested and eligible to join the study.

If you want to learn more and are considering joining the study, please click here [REDCap link]. For more information, please email our study team at [Email address].

Thank you for considering this opportunity. We hope you will join the Daily24 Team!

[Principal Investigator Signature]

Daily24

Principal Investigator: [Principal Investigator Name]
Application Number: [IRB Number]
